# Supplementary figures and images for: Effectiveness and safety of oral lactococci-based vaccine encoding triple common allergens to prevent airway allergy in mice
Source: PLoS One. 2021 Dec 31;16(12):e0261333. doi: 10.1371/journal.pone.0261333 (PMC8719749; doi:10.1371/journal.pone.0261333)

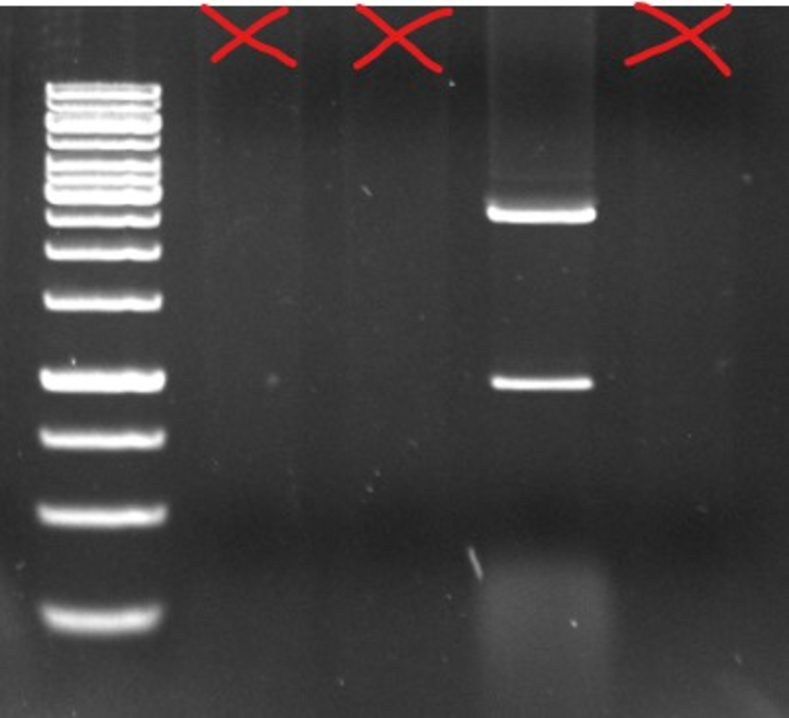

Supplement: S1 Raw images — (ZIP) [file pone.0261333.s001.zip › S-Figure 2-Lane Cla c 14-original gel.tif]

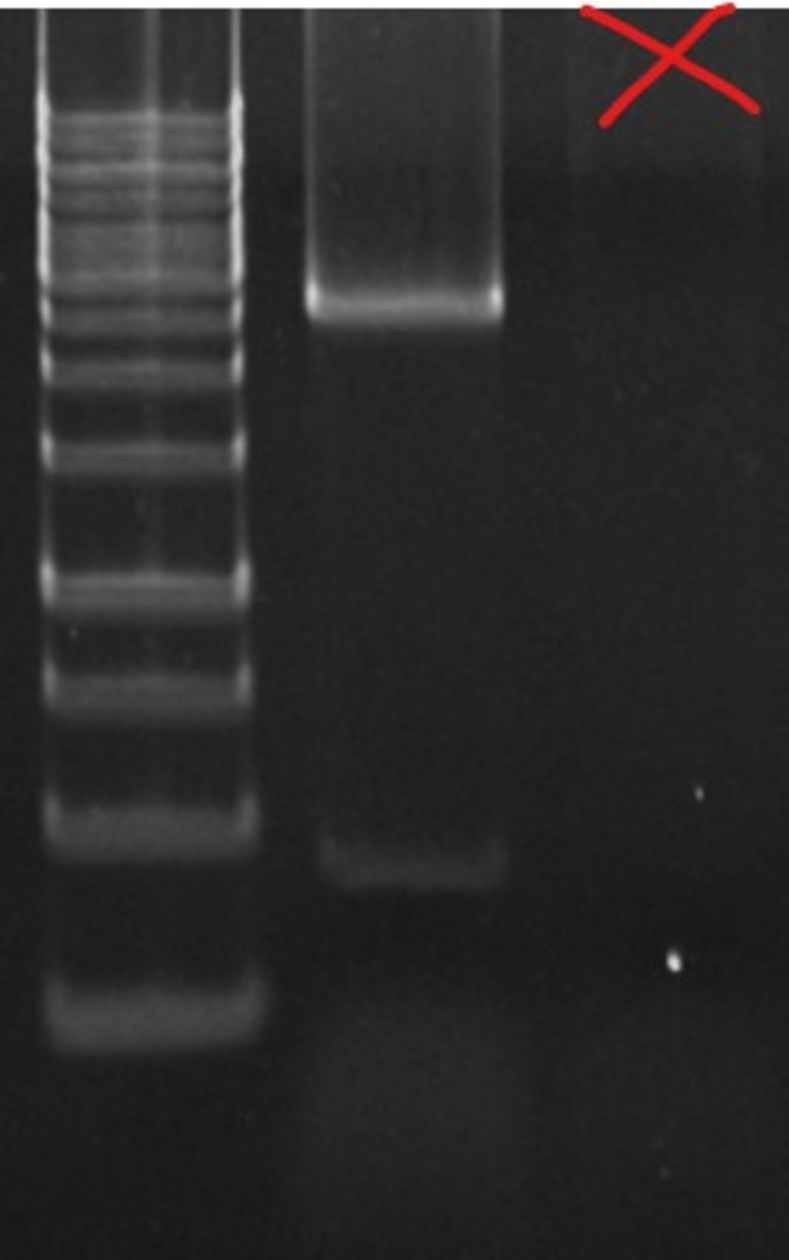

Supplement: S1 Raw images — (ZIP) [file pone.0261333.s001.zip › S-Figure 2-Lane Der p 2-original gel.tif]

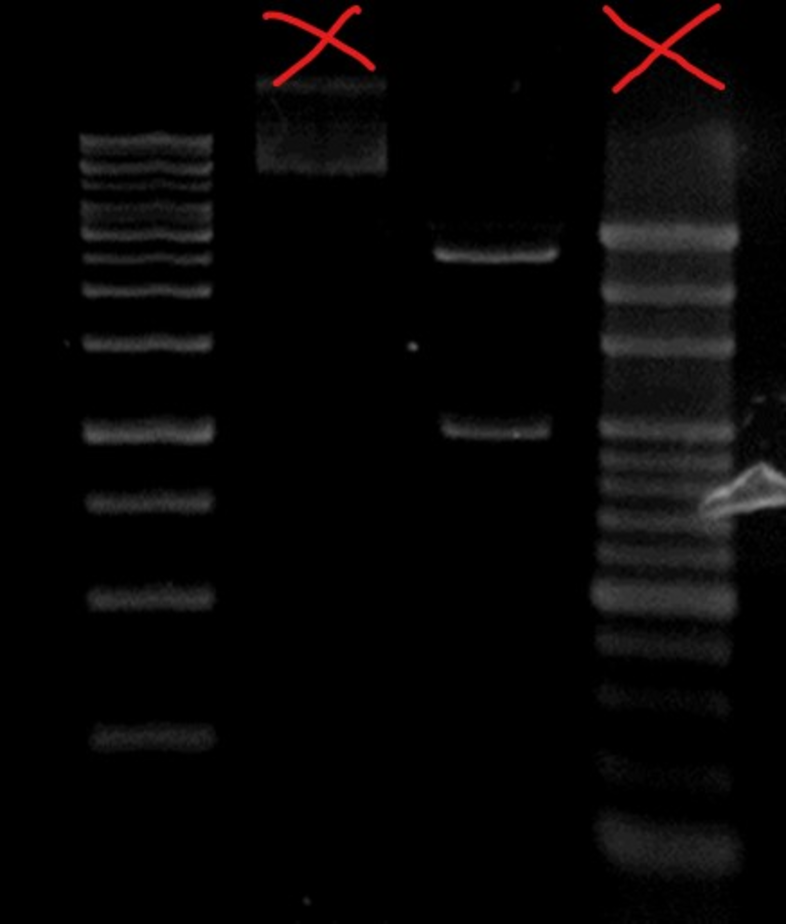

Supplement: S1 Raw images — (ZIP) [file pone.0261333.s001.zip › S-Figure 2-Lane Per a 2-original gel.tif]

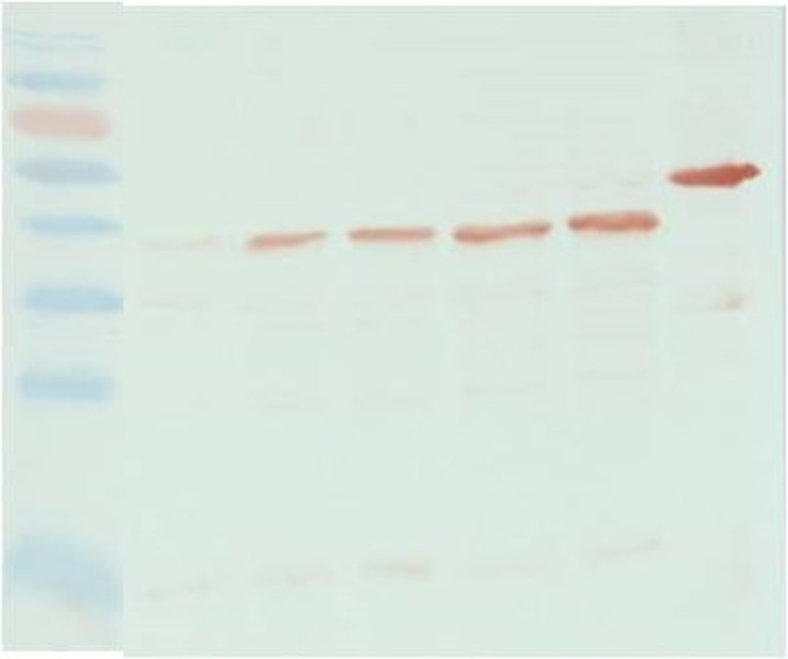

Supplement: S1 Raw images — (ZIP) [file pone.0261333.s001.zip › S-Figure 3A-original blot.tif]

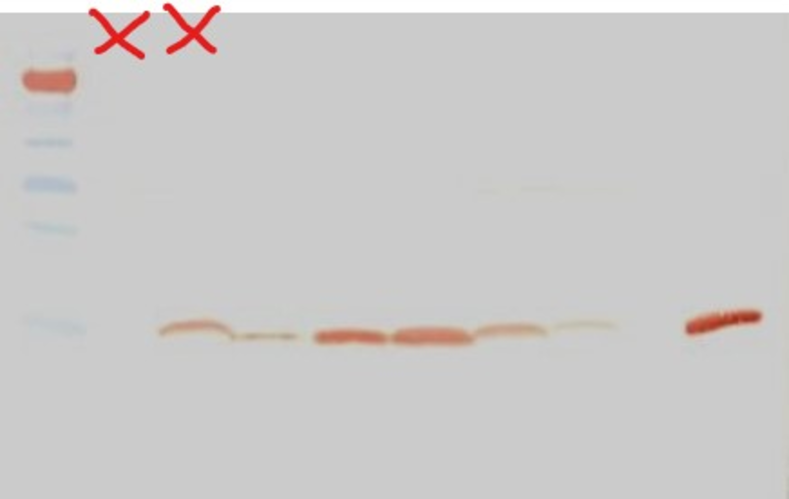

Supplement: S1 Raw images — (ZIP) [file pone.0261333.s001.zip › S-Figure 3B-original blot.tif]

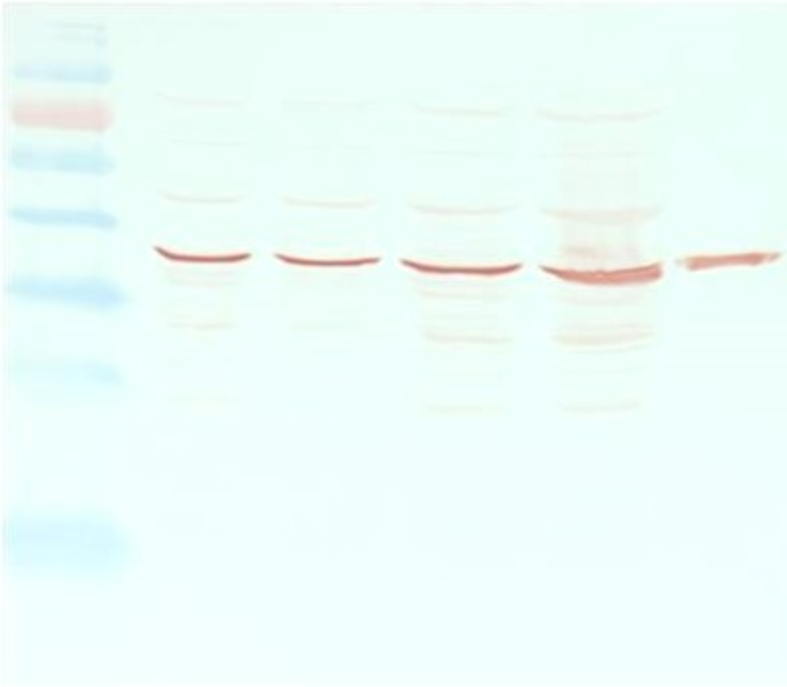

Supplement: S1 Raw images — (ZIP) [file pone.0261333.s001.zip › S-Figure 3Coriginal blot.tif]
